# Mannan-Binding Lectin Suppresses Peptidoglycan-Induced TLR2 Activation and Inflammatory Responses

**DOI:** 10.1155/2019/1349784

**Published:** 2019-01-09

**Authors:** Fanping Wang, Yanhua Li, Can Yang, Yonghui Mu, Yan Wang, Wei Zhang, Yonghui Yang, Chen Chen, Shijun Song, Zhifa Shen, Wenjun Wang, Junpeng Li, Jingjing Zhai, Kang Guo, Ruili Sun, Lili Yu, Mingyong Wang

**Affiliations:** ^1^School of Laboratory Medicine, Xinxiang Medical University, Xinxiang 453003, China; ^2^Collaborative Innovation Center of Molecular Diagnosis and Laboratory Medicine, Xinxiang 453003, China; ^3^School of Basic Medical Sciences, Xinxiang Medical University, Xinxiang 453003, China; ^4^Department of Clinical Laboratory, The Third Affiliated Hospital of Xinxiang Medical University, Xinxiang, China; ^5^State Key Laboratory of Respiratory Diseases, Center for Translational Medicine, The First Affiliated Hospital of Guangzhou Medical University, Guangzhou 510120, China; ^6^Department of Laboratory Medicine, Women and Infants Hospital of Zhengzhou, Zhengzhou 450012, China

## Abstract

Peptidoglycan (PGN), as the major components of the bacterial cell wall, is known to cause excessive proinflammatory cytokine production. Toll-like receptor 2 (TLR2) is abundantly expressed on immune cells and has been shown to be involved in PGN-induced signaling. Although more and more evidences have indicated that PGN is recognized by TLR2, the role of TLR2 PGN recognition is controversial. Mannan-binding lectin (MBL), a plasma C-type lectin, plays a key role in innate immunity. More and more evidences show that MBL could suppress the amplification of inflammatory signals. Whether MBL can alter PGN-elicited cellular responses through TLR2 in macrophages is still unknown, and possible mechanism underlying it should be investigated. In this study, we found that MBL significantly attenuated PGN-induced inflammatory cytokine production, including TNF-*α* and IL-6, in PMA-stimulated THP-1 cells at both mRNA and protein levels. The expression of TLR2 was strongly induced by PGN stimulation. Furthermore, the administration of TLR2-neutralized antibody effectively suppressed PGN-induced TNF-*α* and IL-6 expression. These results supplied the evidence that PGN from *Saccharomyces cerevisiae* could be recognized by TLR2. In addition, we also found that MBL decreased PGN-induced TLR2 expression and suppressed TLR2-mediated downstream signaling, including the phosphorylation of I*κ*B*α*, nuclear translocation of NF-*κ*Bp65, and phosphorylation of MAPK p38 and ERK1/2. Administration of MBL alone did not have an effect on the expression of TLR2. Finally, our data showed that PGN-mediated immune responses were more severely suppressed by preincubation with MBL and indicated that MBL can combine with both TLR2 and PGN to block the inflammation cytokine expression induced by PGN. All these data suggest that MBL could downregulate inflammation by modulating PGN/TLR2 signaling pathways. This study supports an important role for MBL in immune regulation and signaling pathways involved in inflammatory responses.

## 1. Introduction

Infection with Gram-negative as well as Gram-positive bacteria can cause complications of sepsis, lead to septic shock, and result in multiorgan failure and death eventually [1–3]. Peptidoglycan (PGN), also known as murein, is a polymer consisting of sugars and amino acids that forms a mesh-like layer outside the plasma membrane in both Gram-positive bacteria and Gram-negative bacteria [4, 5]. Peptidoglycan consists of *N*-acetylglucosamine (GlcNAc) and *N*-acetylmuramic acid (MurNAc) disaccharide repeating arrays that are cross-linked by short peptides [6]. PGN can elicit the excessive release of proinflammatory cytokines from immune cells, which result in many of the adverse clinical manifestations during bacterial infections [7, 8].

The innate immunity system represents the first line of host defense against invading microorganisms. Pattern recognition receptors (PRRs), which are the most important molecules of the innate immune system, can recognize pathogen-associated molecular pattern (PAMP) molecular structures that are produced by microbial pathogens [9, 10]. Over decades, one of the best identified is the Toll-like receptors (TLRs). To date, thirteen TLRs from mammalian cells and their corresponding ligands have been identified [11, 12]. Among them, TLR2 mediates the host response to Gram-positive bacterium and Gram-negative bacteria, respectively [13]. Certain components of Gram-positive bacterial cell walls, including lipoteichoic acid and lipoproteins, are demonstrated to activate TLR2 [13]. Although more and more evidences have indicated that PGN is recognized by TLR2, the role of TLR2 in cell activation by PGN is controversial with some reports supporting it [14–19] and others are not [20, 21]. Further evidence should be supplied. TLR2 mediates the downstream signaling through the myeloid differentiation factor-8 complex and activates the transcription factors activator protein- (AP-) 1, nuclear factor kappa-B (NF-*κ*B), and mitogen-activated protein kinase (MAPK) [22, 23]. Downstream signaling pathways subsequently lead to proinflammatory responses and cytokine production [24, 25].

Mannan-binding lectin (MBL) is a calcium-dependent lectin of collectin family, which synthesized by the liver and secreted into the blood vessels [26]. It has been considered as a key molecule in innate immunity [27]. MBL plays an important role in the inflammatory response during infection for binding to microbial cell surfaces and activating the lectin pathway of the complement system [28, 29]. MBL is a pattern recognition molecule, specific for a broad spectrum of ligands. It recognizes neutral carbohydrate patterns, such as mannose, glucose, *L*-fucose, *N*-acetylmannosamine, and GlcNAc, which are found on the surface of a large number of pathogenic microorganisms [30].

The role of MBL as a modulator of infection appears to be necessary, and more and more evidences show that MBL could suppress the amplification of inflammatory signals. Accordingly, a study shows that double-stranded RNA-mediated TLR3 activation and innate immunity were attenuated by MBL [31]. MBL and ficolin-A inhibit lipopolysaccharide- (LPS-) mediated proinflammatory responses on mast cells [32]. MBL reduces CpG-DNA-induced inflammatory cytokine production in human monocytes [33]. Monocyte proliferation was inhibited by MBL through transforming growth factor-*β*1 and p38 signaling pathways [34]. In our previous studies, LPS-induced tumor necrosis factor-alpha (TNF-*α*) and IL-12 production were inhibited by MBL in THP-1 cells and monocyte-derived dendritic cells (mDCs) and LPS-induced NF-*κ*B DNA binding and translocation were also suppressed [35, 36]. In addition, we also found that *Candida albicans*-induced cellular inflammation signals mediated by TLR2 and TLR4 were inhibited by MBL [37]. It has been reported that MBL could inhibit PGN-induced proinflammatory effect and promote chemokine production via binding with PGN [6]. However, TLR2 play critical roles in recognition and signaling of PGN and its mechanism of action remains incompletely characterized. More in-depth studies should focus on the mechanism underlying inhibition of MBL in the regulation of PGN-induced inflammatory effect and how MBL regulates TLR2-mediated signaling induced by PGN.

In this study, we demonstrated that MBL suppressed PGN-induced inflammatory cytokine expression in PMA-activated THP-1 cells. The expression of TLR2 was strongly induced by PGN stimulation, and anti-TLR2 antibody blocked inflammatory cytokine that caused by PGN. The expression of TLR2 and TLR2-mediated downstream signaling, including NF-*κ*B activation and phosphorylation of MAPKs, were also attenuated in the presence of MBL. Finally, we found that PGN-mediated immune responses were more severely inhibited by preincubation with MBL. This study supports an important role for MBL involved in inflammatory responses and signaling pathways.

## 2. Materials and Methods

### 2.1. Reagents

Human MBL protein was purchased from ACRO Biosystems (MBL-H5220), and the purity > 95% as determined by SDS-PAGE. PGN was purchased from Sigma (72789) with the purity > 95%, which is isolated from *Saccharomyces cerevisiae*. Anti-MBL polyclonal antibodies (R&D Systems, MN, USA), anti-TLR2 antibody (clone TL2.1, eBioscience, San Diego, CA), and mouse IgG2a were purchased from eBioscience. The specific antibody for I*κ*B-*α*, p65, phospho-I*κ*B-*α*, p38 MAP kinase, c-Jun N-terminal kinase (JNK), phosphorylated ERK1/2, phospho-p38 MAP kinase, phosphorylated JNK and secondary rabbit anti-mouse IgG-HRP antibody were purchased from Cell Signaling Technology (Danvers, MA, USA). Actin, Histone H1, goat anti-rabbit IgG-HRP, and donkey anti-goat IgG-HRP antibody were purchased from Santa Cruz Biotechnology.

### 2.2. Cell Culture

The monocytic human cell line THP-1 was cultured in endotoxin-free RPMI-1640 medium (Gibco, Gaithersburg, MD, USA) supplemented with 10% (*v*/*v*) fetal calf serum (Gibco BRL, Grand Island, CA, USA) and was grown at 37°C in a humidified incubator with 5% (*v*/*v*) CO_2_. Cell viability was valued by trypan blue staining. Firstly, THP-1 monocytes were stimulated with 1 ng/ml phorbol 12-myristate 13-acetate (PMA, Sigma, Saint Louis, MO) for inducing naïve THP-1 cell line differentiation into the active macrophage, for 24 h, and then the activated macrophage was stimulated with PGN (200 *μ*g/ml).

### 2.3. Cytokine Measurements

THP-1 cells (1 × 10^6^/ml) were treated with PMA (1 ng/ml) for 24 h for activation. After washing, the cells were treated with PGN (200 *μ*g/ml) either alone or in complex with MBL (10 *μ*g/ml) that was generated by preincubation for 2 h at room temperature. Stimulated cells were incubated at 37°C in a 5% (*v*/*v*) CO_2_ environment for 24 h. Cell culture supernatants were collected. The levels of IL-6 and TNF-*α* were measured by using IL-6 and TNF-*α* human ELISA kit (R&D Systems, Minneapolis, MN, USA) according to the manufacturer's instructions. Anti-MBL antibody (10 *μ*g/ml) was used for detecting the specificity of MBL and added to mixture containing MBL for 10 min before incubating with the cells.

For determining the priming role of TLR2, PMA-activated THP-1 cells were preincubated with 10 *μ*g/ml anti-TLR2 or with the isotype control of antibody (10 *μ*g/ml) for 1 h, respectively, and then incubated with MBL for 2 h and stimulated with PGN for 24 h. Supernatants were collected from the cells as described above and measured by ELISA.

### 2.4. Reverse Transcription-Polymerase Chain Reaction (RT-PCR) and Quantitative Real-Time PCR (qRT-PCR)

For RT-PCR, total RNA was isolated from treated THP-1 cells by using a TRIZOL reagent (Invitrogen, Carlsbad, CA). The concentration of RNA was detected by NanoDrop microvolume spectrophotometers. Reverse transcription reaction was performed using RevertAid™ First Strand cDNA Synthesis Kit (Fermentas, Glen Burnie, MD, USA) according to the manufacturer's instructions. The primer sequences are shown as follows: human TNF-*α*, 5′-TGGCCTGCACAGTGAAGTGCTG-3′ and 5′-TGGCCAGAACCAAAGGCTCCCT-3′; human IL-6, 5′-AAATTCGGTACATCCTCGACGG-3′ and 5′-GGAAGGTTCAGGTTGTTTTCTGC-3′; human TLR2, 5′-GCCAAGCTCTCTAAACATTT-3′ and 5′-TTTCACTGCTTTCAACTGGTA-3′; and human GAPDH, 5′-CTCCTCCTGTTCGACAGTCAGC-3′ and 5′-CCCAATACGACCAAATCCGTT-3′. Reaction mixtures were prepared, including dNTPs, primers, template cDNA, necessary enzymes, and a buffer solution. The PCR cycling conditions were 95°C for 2 min, followed by 40 cycles of 95°C for 15 s and 60°C for 60 s. PCR products were analyzed by electrophoresis and quantitatively evaluated by densitometry scanning.

Quantitative real-time PCR was performed in the Rotor-Gene 6000 real-time PCR detection system (Qiagen, Germany). Reaction mixtures were completed in a 20 *μ*l volume, including template cDNA, specific primers of each gene, and the platinum SYBR Green SuperMix-UDG (Invitrogen, Carlsbad, CA). Primers specify was analyzed by the relative standard curve. Gene expression was normalized to the expression of GAPDH and quantified by a standard 2^(−ΔΔCt)^ calculation method.

### 2.5. Western Blot Analysis

Cytoplasmic and nuclear proteins were obtained from different treatments of THP-1 cells by Nuclear and Cytoplasmic Protein Extraction Kit (Beyotime Biotech) according to the manufacturer's instruction. Equal amounts of total lysates and nuclear fraction protein were loaded onto a gel, separated by SDS-PAGE, and transferred to a nitrocellulose membrane (Roche). After blocking with 5% nonfat dry milk powder in 1 × Tris-buffered saline and 0.05% Tween 20 (TBST) for 1 h at room temperature, the membranes were incubated with the appropriate primary antibody at 4°C overnight. Then the membranes were washed for three times with TBST and incubated with the appropriate HRP-conjugated secondary antibody for 1 h at room temperature. After washing three times with TBST, the signals were visualized using ECL Plus substrate (Thermo Scientific, Waltham, MA, USA).

### 2.6. Statistical Analysis

The values were presented as mean ± SEM at least three independent experiments and calculated by Excel software (Microsoft). One-way ANOVA was used for comparisons among multiple groups. Student's *t*-test was used for comparisons between two groups. A value of *p* < 0.05 was considered statistically significant.

## 3. Results

### 3.1. MBL Suppresses PGN-Induced Inflammatory Cytokine Expression in PMA-Activated THP-1 Cells

**Figure 1:**
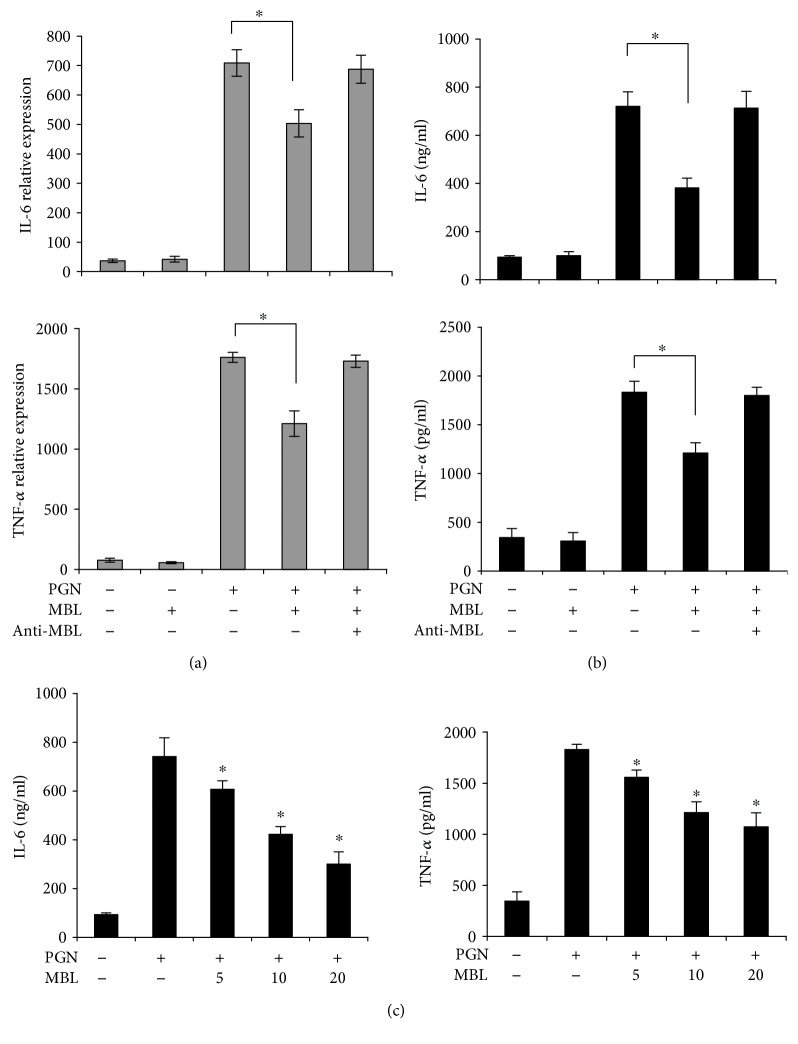
MBL suppresses PGN-induced inflammatory cytokine production. (a) The mRNA levels of TNF-*α* and IL-6. PMA-activated THP-1 cells were stimulated with PGN (200 *μ*g/ml), MBL (10 *μ*g/ml) mixed with PGN, or anti-MBL pAb (10 *μ*g/ml) mixed with MBL and PGN; then the cells were incubated at 37°C in a 5% (*v*/*v*) CO_2_ environment for 24 h. All of the mixture was generated by preincubation for 2 h at room temperature. Total RNA was extracted from the treated cells, and the relative mRNA level of TNF-*α* and IL-6 was analyzed by qRT-PCR by normalizing to internal *β*-actin. Value is mean ± SEM of three experiments. ^∗^*p* < 0.05. (b) The protein levels of TNF-*α* and IL-6. PMA-activated THP-1 cells were stimulated as described in (a). The protein levels of TNF-*α* and IL-6 in the medium were detected by ELISA assays, respectively. Data shown represent three independent experiments with similar results. ^∗^*p* < 0.05. (c) Different concentrations of MBL on PGN-induced cytokine production. PMA-activated THP-1 cells were stimulated with PGN (200 *μ*g/ml) or PGN mixed with 0, 5, 10, and 20 (*μ*g/ml) MBL by preincubation for 2 h in room temperature separately. Total cell medium was collected. The protein levels of TNF-*α* and IL-6 in the medium were detected by ELISA assays. Data shown represent three independent experiments. ^∗^*p* < 0.05.

To determine whether MBL could regulate PGN-induced inflammatory cytokine response, THP-1 cells were stimulated with PMA for macrophage differentiation and then were treated with PGN either alone or in complex with MBL that was generated by preincubation for 2 h at room temperature. The PGN induced highly TNF-*α* and IL-6 production by PMA-activated THP-1 macrophages in a concentration-dependent manner (data not shown). PGN mixed with MBL significantly attenuated the production of TNF-*α* and IL-6 compared with PGN alone as indicated at both mRNA levels (Figure 1(a)) and protein levels (Figure 1(b)). The endotoxin-free MBL did not directly induce TNF-*α* and IL-6 production at PMA-activated THP-1 macrophages compared with the control group (Figures 1(a) and 1(b)). Treatment with MBL antibody during the preincubation of the cells with MBL restored the secretion of TNF-*α* and IL-6, indicating the inhibitory effect caused by MBL (Figures 1(a) and 1(b)). Further MBL also suppressed PGN-mediated TNF-*α* and IL-6 production by PMA-activated THP-1 macrophages in a concentration-dependent manner at protein levels (Figure 1(c)). These results show that MBL suppresses the expression of inflammatory cytokines in response to PGN in the macrophages.

### 3.2. PGN-Induced TLR2 Expression Was Suppressed by MBL

**Figure 2:**
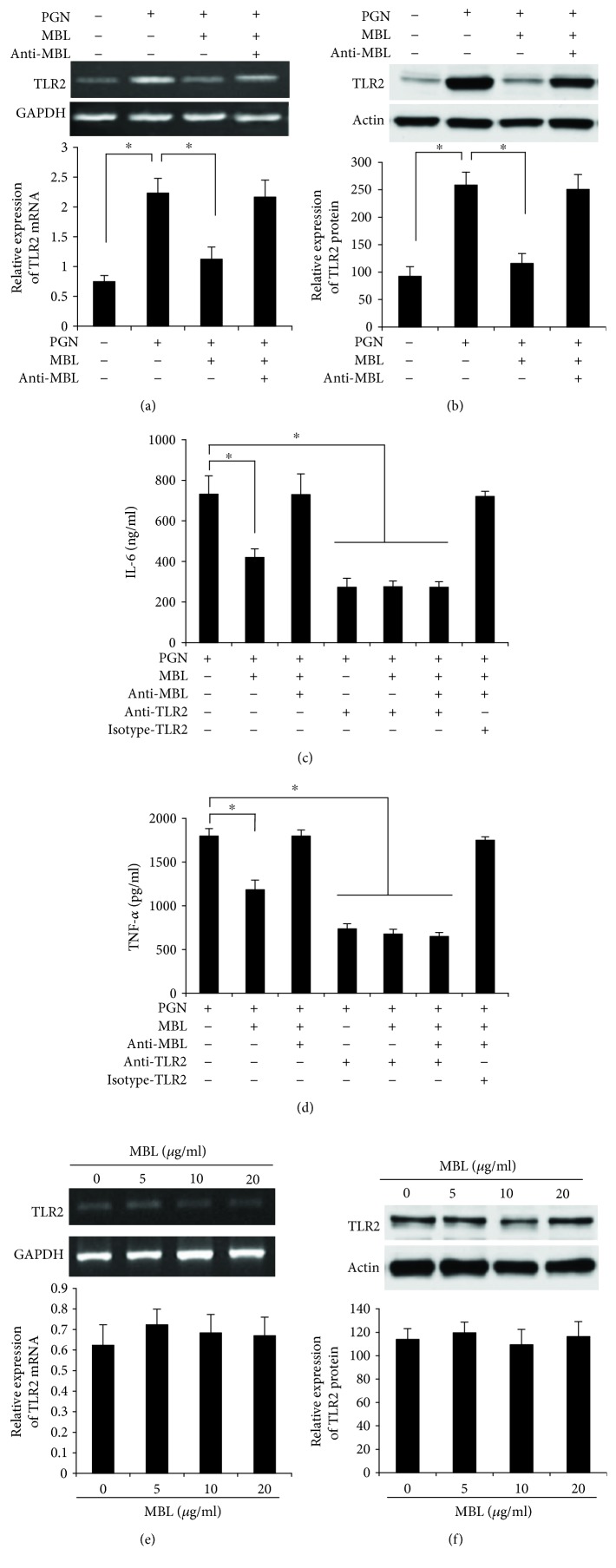
TLR2 is activated by PGN, and the upregulation of TLR2 is inhibited by MBL. (a) The mRNA levels of TLR2. PMA-activated THP-1 cells were stimulated with PGN (200 *μ*g/ml), MBL (10 *μ*g/ml) mixed with PGN, or anti-MBL pAb (10 *μ*g/ml) mixed with MBL and PGN; then the cells were incubated at 37°C in a 5% (*v*/*v*) CO_2_ environment for 24 h. All of the mixture was generated by preincubation for 2 h at room temperature. Cells were harvested, and total RNA was isolated. The mRNA level of TLR2 was analyzed using RT-PCR (upper panel). *β*-Actin was used as loading control. The mRNA levels were quantitatively analyzed based on densitometry analysis (lower panel). (b) The protein level of TLR2. Cells were treated as described in (a), and the protein level of TLR2 was also analyzed using Western blots (upper panel). *β*-Actin was used as loading control. The protein levels were quantitatively analyzed based on densitometry analysis (lower panel). (c) Blocking of TLR2 significantly abolished the inhibitory effect of MBL on IL-6 production. PMA-activated THP-1 cells were pretreated with anti-TLR2 Ab (10 *μ*g/ml) or with the isotype control of antibody (10 *μ*g/ml), respectively, and stimulated with PGN (200 *μ*g/ml), MBL (10 *μ*g/ml) mixed with PGN, or anti-MBL pAb (10 *μ*g/ml) mixed with MBL and PGN. All of the mixture was generated by preincubation for 2 h at room temperature. Then the cells were incubated at 37°C in a 5% (*v*/*v*) CO_2_ environment for another 24 h. Cell culture supernatants were collected and measured by ELISA. (d) Blocking of TLR2 significantly abolished the inhibitory effect of MBL on TNF-*α* production. Cells were treated as described in (c). Cell culture supernatants were collected and measured by ELISA. (e) The expression of TLR2 is not influenced by MBL in the mRNA level. PMA-activated THP-1 cells were stimulated with different concentrations of 0, 5, 10, and 20 (*μ*g/ml) MBL for 24 h, and the mRNA level of TLR2 was detected by RT-PCR (upper panel). *β*-Actin was used as loading control. The mRNA levels were quantitatively analyzed based on densitometry analysis (lower panel). (f) The expression of TLR2 is not influenced by MBL in the protein level. PMA-activated THP-1 cells were stimulated with different concentrations of MBL (0, 5, 10, and 20 (*μ*g/ml)) for 24 h, and the protein level of TLR2 was detected by Western blot (upper panel). *β*-Actin was used as loading control. The protein levels were quantitatively analyzed based on densitometry analysis (lower panel). Images and data are representative of three experiments. ^∗^*p* < 0.05.

TLR2 play an important role for the host to detect and recognize the pathogen and initiate a rapid defensive response [38, 39]. TLR2 mediate recognition of a wide variety of microbial products; however, the recognition of PGN is controversial. Therefore, we sought to investigate whether PGN could be recognized by TLR2 and whether MBL could affect TLR2 expression in PMA-activated THP-1 cells. We stimulated THP-1 cells for 24 h with PGN and mixture (MBL + PGN), and the expression of TLR2 was examined by Western blot and RT-PCR. The data showed that the expression of TLR2 was strongly induced by PGN stimulation at both mRNA (Figure 2(a)) and protein levels (Figure 2(b)), but the expression levels were downregulated to the basal level in the presence of MBL at the concentration of 10 *μ*g/ml (Figures 2(a) and 2(b)).

The above results indicated that TLR2 could be activated by PGN, and next, we further assessed the effect of TLR2 on PGN-induced inflammatory responses. In the incubation of PMA-activated THP-1 cells with anti-TLR2 antibody (clone TL2.1) before treated with PGN, the expression of cytokine TNF-*α* (Figure 2(c)) and IL-6 (Figure 2(d)) was significantly downregulated compared with PGN treatment alone. Normally, after the block of TLR2, the inhibition effect by 10 *μ*g/ml MBL in both IL-6 (Figure 2(c)) and TNF-*α* (Figure 2(d)) cytokine expression was disappeared. These results indicated that TLR2 play a very important role in the recognition of PGN.

We also detected the TLR2 expression after treating with MBL only. Interestingly, TLR2 is not influenced by MBL stimulation in PMA-activated THP-1 cells at both mRNA levels (Figure 2(e)) and protein levels (Figure 2(f)). These results indicated that TLR2 could be activated by PGN and is not influenced by MBL.

### 3.3. Phosphorylation of I*κ*B*α* and Nuclear Translocation of p65 Stimulated with PGN Were Suppressed by MBL in PMA-Activated THP-1 Cells

**Figure 3:**
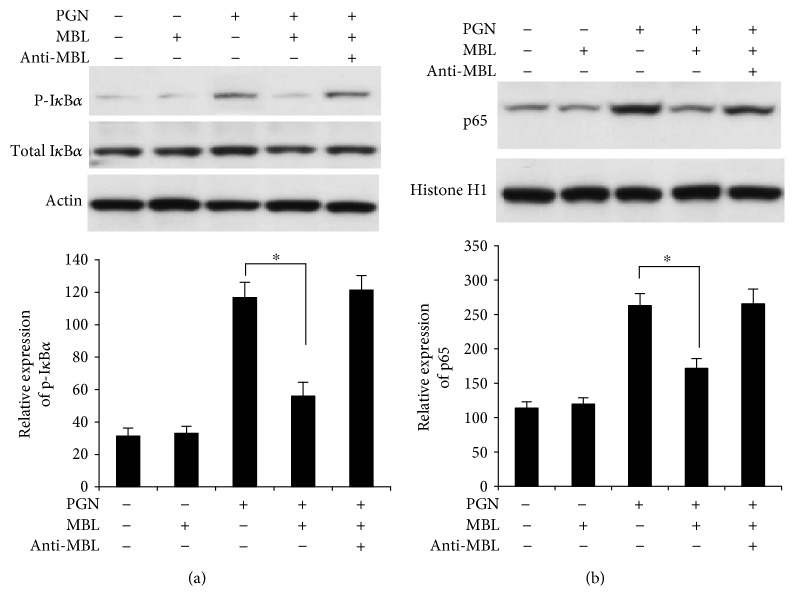
MBL blocks PGN-induced phosphorylation of I*κ*B*α* and p65 nuclear translocation in PMA-activated THP-1 cells. (a) PMA-activated THP-1 cells were stimulated with PGN (200 *μ*g/ml), MBL (10 *μ*g/ml) mixed with PGN, or anti-MBL pAb (10 *μ*g/ml) mixed with MBL and PGN; then the cells were incubated at 37°C in a 5% (*v*/*v*) CO_2_ environment for 8 h. All of the mixture was generated by preincubation for 2 h at room temperature. Cells were harvested, and Western blots were performed using I*κ*B*α* and p-I*κ*B*α* or antibodies (upper panels). *β*-Actin was used as loading control. The protein levels were quantitatively analyzed based on densitometry analysis (lower panel). (b) MBL inhibits p65 nuclear translocation. Cells were stimulated as described in (a). Nuclear extracts were prepared as described in Materials and Methods, and Western blots were performed using p65 antibody (upper panel). Histone H1 was used as loading control. The protein levels were quantitatively analyzed based on densitometry analysis (lower panel). Images are representative of three experiments. ^∗^*p* < 0.05.

We have known that PGN-induced TLR2 expression was suppressed by MBL. It has been known that TLR2 activates the MyD88-dependent pathway and results in the activation of transcription factors NF-*κ*B and AP-1 to induce the expression of cytokine TNF-*α* and IL-6. In order to make sure MBL modulates the downstream signaling of TLR2 induced by PGN, NF-*κ*B activation was detected. I*κ*B*α* has been considered as a key regulator of NF-*κ*B activation, because it can bind to the NF-*κ*B complex in the cytosol to prevent NF-*κ*B translocation to the nucleus and subsequent transcription factor NF-*κ*B-regulated gene expression. NF-*κ*B complex can translocate to the nucleus after the phosphorylation and degradation of I*κ*B*α* [40, 41]. Here, we tested and verified if MBL regulates the phosphorylation of the inhibitory protein I*κ*B*α*. As shown in Figure 3(a), we found that the phosphorylation of I*κ*B*α* in response to PGN after mixing with MBL was decreased compared with treatment with PGN alone in PMA-activated THP-1 cells and MBL did not affect the total protein levels of I*κ*B*α* in these cells.

Having determined that MBL mixed with PGN decreases the phosphorylation of I*κ*B*α*, we further investigate to identify whether MBL could directly regulate the nuclear translocation of the NF-*κ*B complex. We observed that MBL significantly decreased the nuclear translocation of p65, a major component of the NF-*κ*B complex, in the presence of PGN (Figure 3(b)). As shown, the PGN-induced expression of TLR2 was inhibited by MBL; these results verified that TLR2-mediated activation of NF-*κ*B was also suppressed by MBL in THP-1 cells.

### 3.4. Phosphorylation of MAPKs with PGN Was Attenuated by MBL in PMA-Activated THP-1 Cells

**Figure 4:**
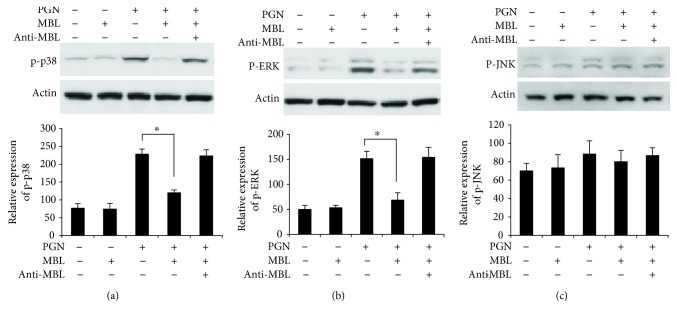
MBL decreases PGN-induced phosphorylation of MAPKs in PMA-activated THP-1 cells. (a) PMA-activated THP-1 cells were stimulated with PGN (200 *μ*g/ml), MBL (10 *μ*g/ml) mixed with PGN, or anti-MBL pAb (10 *μ*g/ml) mixed with MBL and PGN; then the cells were incubated at 37°C in a 5% (*v*/*v*) CO_2_ environment for 8 h. All of the mixture was generated by preincubation for 2 h at room temperature. Cells were harvested, and Western blots were performed using p38 antibody (upper panel). *β*-Actin was used as loading control. The protein levels were quantitatively analyzed based on densitometry analysis (lower panel). (b) Cells were stimulated as described in (a). Western blots were performed using p-ERK (upper panel). The protein levels were quantitatively analyzed based on densitometry analysis (lower panel). (c) Western blots were performed using p-JNK (upper panel). The protein levels were quantitatively analyzed based on densitometry analysis (lower panel). Images are representative of three experiments. ^∗^*p* < 0.05.

Besides NF-*κ*B activation, another TLR2-mediated downstream signaling event is the phosphorylation of extracellular signal-regulated kinase- (ERK-) 1/2, c-Jun N-terminal kinase (JNK), and p38 mitogen-activated protein kinases (MAPKs), which play important roles in cell proliferation, apoptosis, differentiation, cell migration, and gene expression [42]. Several studies have shown that MAPK signaling is also involved in inflammatory response and can activate activator protein-1 (AP-) 1 to induce the expression of TNF-*α* and IL-6 [40, 43]. Thus, we tested to determine whether MBL regulates the phosphorylation of each MAPK in the presence of PGN in PMA-activated THP-1 cells. We found that the phosphorylation of p38 (Figure 4(a)) and ERK-1/2 (Figure 4(b)) was significantly decreased by MBL mixed with PGN compared with PGN treatment alone. However, JNK is not phosphorylated after PGN treatment in PMA-activated THP-1 cells as assayed by Western blot analysis (Figure 4(c)). This means that JNK is not involved in the TLR2-mediated signaling pathway induced by PGN. These results provide further evidence that TLR2-mediated activation of p38 and ERK-1/2 was also suppressed by MBL in THP-1 cells.

### 3.5. PGN-Mediated Immune Responses Were More Severely Suppressed by Preincubation with MBL

Based on our results, we have found that MBL has an effect on TLR2 expression and its downstream signaling pathway. This inhibition occurred on the surface of cell. In our previous study, we supported the evidence for the binding between TLR2 and MBL [37]. According to others' study, MBL could bind with PGN directly [6]. To investigate the possible mechanisms underlying MBL induced inhibition of PGN-mediated immune response, we added the MBL and PGN separately. The first group is negative control; the second group is adding PGN alone; the third group is mixing MBL and PGN for 6 h and adding the mixture to the cell; the fourth group is preincubation of cell with PGN for 6 h and then adding MBL to the cell; the fifth group is preincubation of cell with MBL for 6 h and then adding PGN to the cell.

**Figure 5:**
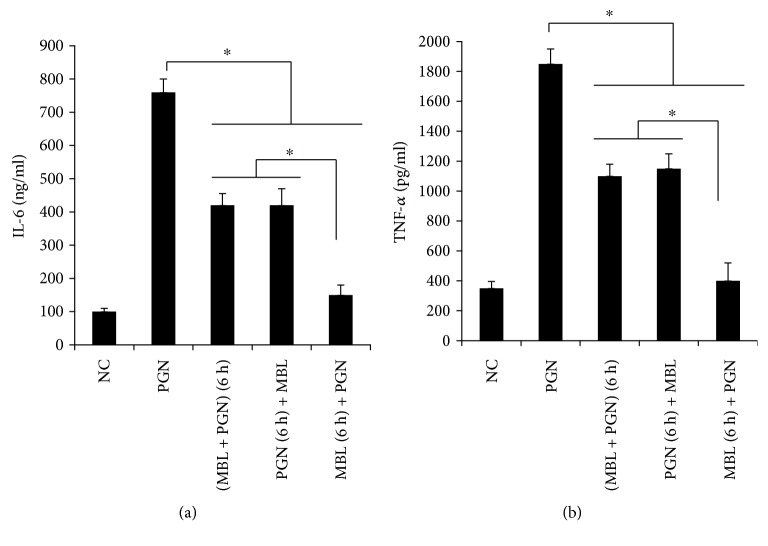
MBL was added before or after treatment with PGN to determine the effect on cellular response. (a) The cytokine levels of IL-6. NC bar indicates a negative control; PGN bar indicates that PMA-activated THP-1 cells were stimulated with PGN (200 *μ*g/ml) alone for 24 h; (MBL + PGN) (6 h) bar indicates preincubation with PGN and MBL (10 *μ*g/ml) for 6 h in room temperature and then were added into PMA-activated THP-1 cells for 24 h; PGN (6 h) + MBL bar indicates that PMA-activated THP-1 cells were preincubated cells with PGN for 6 h and then were added MBL for 24 h; MBL (6 h) + PGN bar indicated that PMA-activated THP-1 cells were preincubated cells with MBL for 6 h and then were added PGN for 24 h. All the cells were incubated at 37°C in a 5% (*v*/*v*) CO_2_ environment. Cell culture supernatants were collected, and IL-6 was measured by ELISA. (b) The cytokine levels of TNF-*α*. Cell culture supernatants from (a) were collected, and TNF-*α* was measured by ELISA. Data shown represent three independent experiments. ^∗^*p* < 0.05.

The results showed that MBL successfully inhibited the PGN-caused inflammation compared with the negative control group in any ways (Figures 5(a) and 5(b)). Interestingly, preincubation with PGN following treatment with MBL has the similar inhibition effect compared with the PGN and MBL mixture group, but the preincubation with the MBL group following treatment with PGN showed a significantly lower level at both IL-6 (Figure 5(a)) and TNF-*α* (Figure 5(b)) compared with these two groups. This indicates that MBL suppressed PGN-induced immune response by blocking the binding between TLR2 and PGN.

## 4. Discussion

Bacterial components such as PGN recognized by the mammalian innate immune system promote host defense against bacterial infection [44, 45]. The innate immune system senses intact PGN and its fragments using PRRs. One of the best studies PRRs for recognizing PGN is TLR2, but the interaction between these two is confusing. Early studies identified PGN as a TLR2 ligand [19, 46]. However, a study in 2004 questioned the TLR2-mediated PGN reaction because of the contamination of lipoproteins and lipoteichoic acids [20]. Soon after, soluble PGN was purified by a group and countered the study of 2004, which can activate a TLR2 reporter cell line [47]. But another study shows that PGN from *Bacillus anthracis* requires internalization and degradation to cause cytokine induction, suggesting that there is no sensor at cell surface for the recognition of PGN [48]. Recently, a review indicated that the variation in peptidoglycan structure from species to species may both contribute to the different results [45].

PGN from *Saccharomyces cerevisiae* was used for our study, and the results indicated that PGN successfully induced the expression of cytokine TNF-*α* and IL-6, overexpression of TLR2, activation of NF-*κ*B, and phosphorylation of MAPKs in PMA-activated THP-1cells. Importantly, PGN-caused secretion of cytokines TNF-*α* and IL-6 were successfully blocked by TLR2 antibody treatment. These results support the evidence that PGN from *Saccharomyces cerevisiae* could be recognized by TLR2 and induced the downstream signaling pathway. From another standpoint, TLR2 antibody did not suppress cytokine expression to basal levels and there are still other PRRs involved in the recognition of TLR2. Studies have shown that nucleotide-binding oligomerization domain-containing protein 1 (NOD1) and NOD2 which belonged to cytosolic NOD-like receptors are the sensors of PGN, which are expressed by diverse cell types [45].

MBL is a major recognition receptor and a circulating C-type lectin in the innate immune system, which is secreted into the bloodstream by hepatocytes [49]. The immune response will be amplified by proinflammatory cytokine secretion, including TNF-*α*, IL-1, and IL-6, which help to enhance the tissue permeability and recruit more phagocytes to clear the bacterial infection [44, 50]. However, proinflammatory cytokine excess will result in septic shock and multiple organ failure [51]. MBL could regulate the inflammatory signals mediated by a variety of microbes and limits TLR signaling so that the host is not damaged by an overreactive inflammatory response [52, 53]. Here, we investigated how the soluble MBL regulated PGN-induced immune responses. In our study, human PMA-activated THP-1 macrophage was selected as a model for studying the role of MBL in immune regulation in vitro similar to our previous studies [35, 37]. THP-1 derived from a patient with acute monocytic leukemia lost some function of macrophage [54] and were stimulated with PMA to induce the naïve macrophage differentiation.

Our study demonstrated that MBL regulated PGN-induced inflammatory cytokine secretion. The data showed that MBL (10 *μ*g/ml) could inhibit PGN-induced inflammatory cytokine (IL-6 and TNF-*α*) production at both mRNA and protein levels in macrophages, suggesting that MBL could inhibit PGN-induced inflammatory responses. These results are consistent with others' result [6]. We found that the overexpression of TLR2 induced by PGN was also decreased by MBL. TLR2-mediated downstream signaling was also detected to verify the function of MBL. NF-*κ*B, as a key transcription factor of lymphocytes and macrophages, is a heterodimer composed of p50 and p65 subunits [55]. NF-*κ*B is characterized by its inactivation in the cytoplasm by binding to I*κ*B kinase, and upon stimulation, it leads to rapid translocation into the nucleus for the degradation of I*κ*B kinase [56]. Our results showed that the phosphorylation of I*κ*B*α* in response to PGN is decreased in MBL-treated macrophage, while the level of total I*κ*B*α* protein in these cells is unchanged compared with the control. In addition, the nuclear translocation of NF-*κ*Bp65 was significantly decreased by MBL treatment. These experiments clarify the regulation role of MBL on the TLR2-mediated NF-*κ*B signaling pathway.

MAPK signaling is another TLR2-mediated downstream pathway [57]. Conventional MAPKs include the ERK1/2, JNK, and the p38 isoforms [58]. Here, we sought to investigate the phosphorylation of each MAPK in the presence of PGN in PMA-activated THP-1 cells. Our results showed that PGN induced the phosphorylation of p38 and ERK, but it did not affect the JNK activation based on Western blot analysis. Another agonist of TLR2, biglycan, also leads to the rapid activation of p38 and ERK in macrophage [59]. However, the agonist Pam3CSK4 could induce ERK1/2, JNK, and p38 phosphorylation in human mast cell [60]. This difference of TLR2-mediated MAPK activation may be due to cell type variation. In addition, similar to NF-*κ*B, the activation of p38 and ERK1/2 caused by PGN was also suppressed by MBL treatment. These experiments clarify the role of MBL on the TLR2-mediated MAPK signaling pathway.

In this study, we showed that MBL could suppress TLR2-mediated signaling pathway induced by PGN, including the expression of TLR2, as well as downstream signals such as NF-*κ*B, MAPKs, TNF-*α*, and IL-6. More and more studies focus on the interactions between TLRs and MBL, and the inhibition function by MBL is consistent with ours. Shimizu et al. showed that recombinant MBL could bind to TLR4 and MD-2 through the carbohydrate recognition domain [61]. Liu et al. reported that MBL could attenuate double-stranded RNA-mediated TLR3 activation and subsequent inflammatory cytokine production [31]. MBL reduces CpG-DNA-induced TLR9 activation and inflammatory cytokine production in human monocytes [33]. We previously demonstrated that MBL could inhibit LPS-induced TLR4 signaling inflammatory response and *C. albicans*-induced TLR2 and TLR4 signaling [35–37].

Next, we focus on the mechanism underlying MBL-suppressed TLR2-mediated innate immune responses induced by PGN, and our data has indicated that this inhibition by MBL has occurred at the cell surface. A study by Nadesalingam et al. has referred that MBL inhibited PGN-induced production of proinflammatory cytokines through binding with PGN [6]. We proved that MBL could bind to the extracellular domain of TLR2 by coimmunoprecipitation experiments [37]. What is the interaction between MBL, PGN, and TLR2? Therefore, we added MBL before and after adding PGN to PMA-activated THP-1 cells to determine if there is a significant difference in the activation of cellular responses. Preincubation of THP-1 cells with PGN 6 h and then adding MBL could block the inflammation cytokine expression by PGN, and this group showed similar cytokine levels compared with mixed PGN and MBL for 6 h and adding mixture to cells (Figures 5(a) and 5(b)). Added MBL after adding PGN still inhibited the cytokine expression compared with the negative control. Furthermore, preincubation of THP-1 cells with MBL 6 h and then adding PGN showed a significant low level compared with the PGN and MBL mixing group (Figures 5(a) and 5(b)).

Based on the above results, there are three assumptions. The first hypothesis is that MBL is only combined with PGN, which block the binding of PGN to TLR2. If this assumption is true, the cytokine levels in preincubation with the MBL group should be similar or higher than the mixing group. MBL and TLR2 will compete against the binding with PGN in this hypothesis. But our results showed that the cytokine levels are lower than the mixing group. Therefore, the first assumption is incorrect. The second hypothesis is that MBL is direct binding to TLR2. If this assumption is true, the cytokine levels in preincubation with the PGN group should be higher than the mixing group. MBL and PGN will compete against the binding with TLR2 in this hypothesis. But our results showed that the cytokine levels in preincubation of PGN are similar with the mixing group. Therefore, the second assumption is not right. The third hypothesis is that MBL can bind with both PGN and TLR2, and our data was conforming to this assumption. In the preincubation with the PGN group, MBL combined with PGN executed a more important role in the downregulation of cytokine induced by PGN. In the preincubation with the MBL group, MBL combined with TLR2 carried out a decisive role in the inhibition of cytokine induced by PGN.

The data has shown that MBL-mediated attenuation of PGN-induced inflammatory responses was abolished with the TLR2 antibody treatment (Figures 2(c) and 2(d)). Interestingly, there is still low levels of IL-6 and TNF-*α* detected from PGN-stimulated macrophages after TLR2 blockade. Indeed, there are other sensors involved in PGN-induced inflammatory responses as we mentioned before that are NOD1 and NOD2. As cytosolic sensors, NOD1 and NOD2 must either detect bacteria that enter the cytosol or peptidoglycan must be degraded to generate fragments that must be transported into the cytosol for these sensors to function [45]. PGN has long been recognized as an important PAMP that is detected by innate immune mechanisms during bacterial infection. Therefore, the present study strongly nominates and recommends the potential usefulness of MBL against bacterial infections and its potential extension at clinical application as a therapeutic agent.

Taken together, this study is to investigate the role of MBL on the regulation of PGN/TLR2 signaling. Our data showed that MBL could suppress PGN-induced inflammatory responses through the TLR2 receptor p38, ERK1/2, and NF-*κ*B pathways. MBL can combine with both TLR2 and PGN to block the inflammation cytokine expression by PGN. This study supports that MBL, as an acute-phase protein, play an important role in the regulation of inflammatory responses and balancing the homeostasis.

## Data Availability

The data used to support the findings of this study are available from the corresponding author upon request.
